# Supplementary material for: Vertical integration selection of Chinese pig industry chain under African swine fever - From the perspective of stable pig supply
Source: PLoS One. 2023 Feb 23;18(2):e0280626. doi: 10.1371/journal.pone.0280626 (PMC9949627; doi:10.1371/journal.pone.0280626)
Supplement: Attachment — Submitted filename: manuscripts.docx [file pone.0280626.s002.docx]

**Vertical Integration Selection of Chinese Pig Industry Chain under African Swine Fever - From the Perspective of Stable Hog Supply**

Gangyi Wang^1^, Jingjing Wang^1*^, Siyu Chen^1,^ Change Zhao^1^

^1^ College of economics and management, Northeast Agricultural University, Harbin, Heilongjiang, China

*Corresponding author:

E-mail: [wangjingjing1115@neau.edu.cn](mailto:wangjingjing1115@neau.edu.cn)

**Abstract:**

Vertical integration is conducive to the realization of complementary interests and sustainable development of pig industry. The outbreak of African swine fever (ASF) in 2018 has disrupted many activities along pig industry chain in China. The production capacity of breeding pigs has dropped rapidly, and the supply of hog is tight. The vertical integration of hog industry chain is the main driving force to ensure food supply. Based on the data of 12 listed pig companies from 2012 to 2019, we examines the ways and reasons for vertical integration of pig companies when external shocks increase by taking ASF as an example, and breakpoint regression and Tobit model are used to analyze differences and determinants of the forward and backward integration of hog industry chain under ASF. The empirical results showed that the forward integration of the feed processing link and slaughter circulation link is higher than the backward integration. ASF had different effects on the vertical integration degree of each link. ASF promoted the forward integration, and the factors affecting the forward integration of hog industry chain had increased. The main factors have different influences on the vertical integration of the China's pig industry. Forward integration increased mainly depends on the income uncertainty, legal system environment, market demand, expenditure uncertainty, transaction frequency. The findings of the study imply that hog industry chain is taking the forward integration to cope with the ASF shock. The combination of feed link and breeding link reduces feed cost and ensures hog supply. Pig companies tend to reduce transaction costs by strengthening the control of downstream supply.

**KEY WORDS:** Pig industry chain; Vertical integration; African swine fever (ASF); Food supply; Listed pig companies;

**1. Introduction**

The hog industry is an important industry related to the national economy and people's livelihood in China. Pork production and consumption in China rank first in the world. The hog industry in China develops rapidly on the whole, but the development of the hog industry chain before, during, and after production links is unbalanced. Some events impact the sustainable development of the hog industry during different periods. Any link affected by external shocks will lead to huge fluctuations in the industrial chain. With the rapid expansion of the overall scale of breeding and the improvement of breeding density, the threat of major diseases to the pig industry is also increasing. The outbreaks of African swine fever (ASF) in China affected many links in the hog industry chain. Although fewer than 200 outbreaks of ASF have occurred, the hog industry still faces profound challenges. The industrial chain is decoupled, the feed supply is seriously lagging, and it is difficult to guarantee the supply in breeding. There are "no pigs to slaughter" and "no pigs to transport" in slaughter and circulation. The hog industry has suffered huge losses. Starting in the third quarter of 2018, breeding sows have recorded a month-on-month decline of more than 1% for 10 consecutive months. Month-on-month change rates since 2019 have fallen by more than 10 percent, and the number of breeding sows in October was about 20.7 million, which was 37.92% lower than that in 2018. Pork consumption accounts for 63% of meat consumption in China.

Low industrial concentration is the underlying cause of these problems, and it also brings great obstacles to solving these problems. Vertical integration of companies' internal industrial chain has obvious comparative advantages in risk response. Spain, which has a similar pig industry structure to China, faced the same pig undersupply decades ago. There are low-scale and industrial concentrations of the hog industry in Spain. The number of slaughters fell under ASF. With the impetus of the Spanish government, the hog industry began to accelerate the development of vertical integration. Feed processing subjects take the initiative to develop backward integration into pig breeding and sign contracts with breeding subjects to achieve stable transactions, so the industrial concentration is further improved, and the scale is also rising rapidly. During this period, the number of scatter-feed households dropped by 110,000, and the number of slaughters on single farms increased from 122 to 467. As of 2017, the hog production of large-scale hog industry chains with vertical integration accounted for 65% of hog production in Spain.

In 2021, the "Opinions of the Ministry of Agriculture and Rural Affairs, the National Development and Reform Commission, the Ministry of Finance, the Ministry of Ecology and Environment, the Ministry of Commerce, and the China Banking and Insurance Regulatory Commission on Promoting the Sustainable and Healthy Development of the Hog Industry" pointed out that it is necessary to improve the stable production and supply of pigs. As the most populous country in the world, pork is the largest animal product in the food consumption of Chinese residents, accounting for 31% of the total animal food consumption. The pork supply is an important guarantee for food supply in China. ASF has shocked the hog industry chain and exposed pork supply to risks. From the perspective of practice, pork supply needs to be completed by many companies. Listed pig companies occupy a prominent position in the development of the industry, which is mainly manifested in market concentration and product recognition, as well as their impact on the development of the hog industry and industrial policies. Vertical integration can increase the market concentration of companies and ensure food supply. Previous studies of hog industry chain have focused on pig breeding [1, 2]. When companies as a member of the chain [3, 4], it can significantly affect the performance of the company and industrial chain competitiveness [5-7].

ASF has disrupted hog industry chain [8], and the impacts on food supply even extend upward to the upstream production part of hog industry [9]. As a highly contagious disease, on the one hand, ASF has caused volatility hog market and hindered the sustainable and high-quality development of the hog industry in China [10-12]. ASF threatens food supply and challenges the livelihoods of pig producers and other actors along the supply chain [13], and the total pig herd and pork production volume has decreased [14]. The drastic reduction in pork supply has led to a sharp increase in pork prices [15]. On the other hand, ASF has transformed hog industry in China into medium and large-scale farms, as well as better-standardized production systems and biosecurity [16]. It can be seen that ASF has an impact on all aspects of hog industry chain, so it is necessary to find a way to ensure food supply.

Vertical integration is widely used by researchers because of its essential role in improving production and strengthening the connection between upstream and downstream of the industry chain [17, 18]. Although some studies have shown that vertical integration reduces production [19], most studies suggest that vertical integration can improve the performance of farmers [20]. Participation in written and verbal contracts in a vertical integration mechanism can increase the quality of the relationship between farmers and buyers, and positively affects the performance of farmers [21]. In addition, some countries increase hog industry chain value through vertical integration[22, 23], and vertical integration also effectively prevents disease in the hog industry [24]. Vertical integration can improve the performance of the hog industry, alleviate the impact of the epidemic on the industry chain, and ensure a stable food supply.

Previous studies provide a solid foundation for this paper. However, hog industry chain vertical integration needs to be further studied: First of all, the vertical integration of the industrial chain can improve the competitiveness of enterprises. The previous studies on the industrial chain focus more on the performance or efficiency of the supply chain operation and have never explored what factors affect the vertical integration of the industry chain. Secondly, previous studies did not consider the impact of the epidemic on the vertical integration of the hog industry chain. In theory, the epidemic will change the behavior of all players in the industrial chain. It is necessary to explore the influence of ASF on the vertical integration of the industrial chain. At present, vertical integration of the hog industry chain will be an important direction to ensure food supply under the normalization of ASF in China. Ether dealing with ASF or ensuring food supply, the most important solution is to break the decoupling status of the hog industry chain. The vertical integration development promotion of the industry chain can stabilize the food system from the supply side. Therefore, this paper firstly analyzed the influence mechanism of transaction costs on the vertical integration of industrial chain from the theoretical level, and then discussed the vertical integration degree of hog industry chain in China, the integration degree of each link, the obvious difference of transaction costs faced by the pig enterprises in different links and the determinants of vertical integration. Taking ASF as an example, This paper will explore the ways and reasons for vertical integration of pig enterprises when external shocks increase.

**2. Theoretical framework**

The concepts of industry chain and supply chain are essentially consistent, aiming to coordinate the behaviors of various entities in the chain, improve the overall operating efficiency of the chain, and promote maximum utility. We define hog industry chain as a network structure composed of all functional links that have upstream and downstream relations with pig breeding under the relevant legal environment and resource environment. Hog industry chain is an open system, and the subjects independently select transaction models based on their interest relationships to improve their benefits. The external environment is constantly changing, which disturbs the organizational mode and transaction rules of the constituent elements in the chain.

The essence of industry chain integration is that enterprises break through boundaries, seek the best combination of resources in the industrial chain, so as to reduce transaction costs, amend resource constraints by expanding the available resource space, and realize the transformation of resources from narrow sense to broad sense and from outside to inside.

Economists have explained the reasons of vertical integration from different perspectives. The vertical integration refers to the realization of a win-win situation for upstream and downstream enterprises to improve efficiency and reduce costs through the optimization of the value chain, supply and demand chain and space chain. As early as 1937, Coase proposed that vertical integrate depends on the size of transaction costs. In order to avoid opportunistic behaviors in transactions and reduce transaction costs, enterprises will choose vertical integration [25], and replace commodity contracts of transaction products with labor factor contracts [26].

Asset specificity, uncertainty, and transaction frequency are three key factors that affect transactions [27]. If the special assets invested in the transaction are misappropriated, the value of the assets will be reduced, and business operations will be affected [28]. When the specific assets of the company are relatively high, the operational losses can be reduced by adopting closer vertical cooperation. The theory of industrial organization further points out the key to vertical integration is maintaining the control of upstream and downstream businesses. Scope economy promotes the synergy of the industry chain, and downstream companies have strong monopoly power and have greater control over the pricing power of upstream companies. Vertical integration expands the scale of enterprises [29], reduces the risk of opportunism by internalizing the trading market, increases the profits of the industry chain [30], and re-allows monopoly benefits [31].

When the degree of uncertainty and risk is high, diversified business operations can offset some of the risks and protect the long-term survival and development of the enterprise. The real options theory points out that uncertainty is the source of income, not risk and threat [32]. Vertical integration can avoid market risks and reduce the uncertainty of enterprise production and sales [28, 33].

The more frequent the transaction is, the more transaction costs are. It is necessary to establish a closer collaboration method to reduce transaction costs. At the same time, the cost of establishing such a dedicated governance structure will be easily compensated [28]. From the perspective of endogenous competitive advantage, the theory of enterprise capability believes that vertical integration can better combine complementary skills inside and outside the enterprise, produce management and operating synergy, and improve production efficiency [34].

Institutional environment theory analyzes the impact of vertical integration from the external institutional level of the industry chain. When the legal environment in the area where the company is located is better, the risk of default when the company signs a contract and the cost of contract execution will be reduced. When the environment and investor protection are poor, companies will merge the production originally organized by the market into the company, thus saving transaction costs and reducing corporate risks [35]. The uncertainty brought by changes in the policy environment affect the vertical integration of enterprises [36].

Based on the previous theoretical studies, we starts from the on-chain and off-chain of the industry chain, with transaction cost theory and institutional environment theory as the main theoretical framework, focusing on the analysis of transaction costs and institutional environment on the vertical integration of hog industry chain while considering other factors.

**Fig. 1. Theoretical framework**

**3. Vertical integration calculation and comparative analysis of hog industry chain**

**3.1. The level of vertical integration**

The I-O method (input-output method) is used to calculate the integration index of the subject of hog industry chain by using the market share data and the input-output relationship.

Indicators for input-output method are constructed. According to the end-of-year and mid-year reports of the subject, among the business incomes classified by industry, the industry with the highest proportion of business income is positioned as the main department $i$, and the other business income industries $j$ are the auxiliary department of the subject of hog industry chain. Through the input-output table issued by the National Bureau of Statistics in 2018, the input $\vartheta_{ij}$of $j$ per unit output required for $i$ is calculated, as is the input $\vartheta_{ij}$ of $j$ per unit output inflow to $i$, ${Mean}_{ij, imput}=\frac{\vartheta_{ij}+\vartheta_{jt}}{2}$ . This variable represents the integration opportunity of the main department $ı$ and the auxiliary department $j$. $\gamma_{j}$is the income ratio of the auxiliary department $j$ to the total income of the non-main department.

Backward Integration Degree Model:

$V_{backward}=\sum r_{j}{Mean}_{ij,imput}$	(1)

Forward Integration Degree Model:

$V_{forward}=\sum r_{j}{Mean}_{ij,imput}$	(2)

Table 1. Statistical description

|  | Max. | Min. | Avg. |
| --- | --- | --- | --- |
| ${Mean}_{ij,imput}$ | 0.34 | 0.01 | 0.16 |
| ${Mean}_{ij,imput}$ | 0.34 | 0.01 | 0.17 |
| ${Income}_{total}$ | 72,526,310,239.06 | 214,104,119.26 | 13,464,032,767.95 |
| ${Income}_{t}$ | 57,557,623,514.21 | 221,178.26 | 2,880,825,559.80 |
| ${Income}_{forward j}$ | 22,577,165,714.29 | 2,475.00 | 991,398,663.71 |
| ${Income}_{backward j}$ | 1,594,575,386.58 | 12,688.27 | 203,505,802.52 |

**3.2. Data selection**

According to the main business income, the enterprises with the highest operating income of pig-related products were selected. Based on this standard, there are 25 listed pig companies. To ensure the continuity of main business data from 2012 to 2019, 4 listed companies were selected for each link in the end. The annual reports and 2018 national input-output table of 12 listed pig companies were used as data sources. The listed pig companies selected cover the three links of feed, breeding and slaughter in hog industry chain. With the form of "company + farmer", they lead farmers to connect to the market, gradually increase their market share, and form an industrial layout all over the country, covering more than 70% of pig breeding and more than 90% of pork supply. In Feed processing, the listed companies we selected were TRS, TKSW, ZBKJ, and JXN. Pig breeding were WSGF, MYGF, TBGF, CJNM, and Slaughter circulation were XWF, SHFZ, LDRS, and HTGF. In addition to the pig breeding business, these companies have also affected 370 million pigs in China through feed sales, gene promotion or cooperative breeding (the number of pigs for slaughter in 2020 is 522.04 million). Among them, 12 listed companies have more than 30 national-level core pig farms and more than 40 cooperative breeding.

**3.3. Comparative Analysis on Vertical Integration of Hog Industry Chain**

The vertical integration of hog industry chain in China showed an upward trend, and the degree of forward integration was higher than that of backward integration (Table 2). It means that under the guidance of relevant policies in China, hog industry chain continuously has been developing vertical integration of industry according to the characteristics of the development of the hog industry. Through this development model, the combination of upstream and downstream industries was realized to reduce the transaction cost of the subject of hog industry chain.

Table 2. The degree of vertical integration in hog industry chain in the first half year, the second half and the whole year 2012-2019 (%)

| Year | | | 2012 | 2013 | 2014 | 2015 | 2016 | 2017 | 2018 | 2019 |
| --- | --- | --- | --- | --- | --- | --- | --- | --- | --- | --- |
| Feed processing | Backward | H1 | - | - | - | 0.91 | 0.27 | 0.06 | 0.02 | 0.1 |
|  |  | H2 | - | - | - | 13.79 | 52.6 | 62.07 | 62.94 | 70.78 |
|  |  | yearly | 1.53 | 0.86 | 0.93 | 0.89 | 0.41 | 0.12 | 0.12 | 0.09 |
|  | Forward | H1 | - | - | - | 39.94 | 30.96 | 56.12 | 54.82 | 60.43 |
|  |  | H2 | - | - | - | 0.87 | 0.46 | 0.17 | 0.21 | 0.08 |
|  |  | yearly | 31.99 | 40.75 | 42.05 | 34.68 | 43.86 | 61.55 | 59.76 | 67.51 |
| Pig breeding | Backward | H1 | - | - | - | 31.12 | 31.12 | 17.1 | 66.95 | 60.06 |
|  |  | H2 | - | - | - | 1.71 | 2.21 | 4.14 | 5.08 | 6.87 |
|  |  | yearly | 35.23 | 31.66 | 32.14 | 36.76 | 32.92 | 37.75 | 35.79 | 39.04 |
|  | Forward | H1 | - | - | - | 1.72 | 2.92 | 1.07 | 9.21 | 3.42 |
|  |  | H2 | - | - | - | 18.45 | 17.44 | 16.22 | 22.23 | 38.29 |
|  |  | yearly | 0 | 0 | 1.5 | 1.78 | 3.81 | 4.84 | 5.8 | 5.34 |
| Slaughter circulation | Backward | H1 | - | - | - | 21.7 | 18.76 | 17.43 | 11.45 | 0.17 |
|  |  | H2 | - | - | - | 41.84 | 37.27 | 37.52 | 57.61 | 46.37 |
|  |  | yearly | 4.23 | 3.86 | 3.1 | 3.95 | 3.19 | 2.57 | 0.79 | 0.52 |
|  | Forward | H1 | - | - | - | 37.84 | 38.02 | 41.1 | 39.1 | 56.92 |
|  |  | H2 | - | - | - | 21.13 | 17.97 | 15.34 | 8.22 | 12.14 |
|  |  | yearly | 31.72 | 31.63 | 35.91 | 35.34 | 35.12 | 35.23 | 42.6 | 41.45 |
| Avg. | Backward | H1 | - | - | - | 17.91 | 16.72 | 11.53 | 26.14 | 20.11 |
|  |  | H2 | - | - | - | 19.11 | 30.69 | 34.58 | 41.88 | 41.34 |
|  |  | yearly | 13.66 | 12.12 | 12.06 | 13.87 | 12.17 | 13.48 | 12.24 | 13.22 |
|  | Forward | H1 | - | - | - | 26.5 | 23.97 | 32.76 | 34.38 | 40.26 |
|  |  | H2 | - | - | - | 13.48 | 11.96 | 10.58 | 10.22 | 16.84 |
|  |  | yearly | 21.23 | 24.13 | 26.4 | 23.93 | 27.6 | 33.87 | 36.05 | 38.1 |

Note: H1 indicates the first half year, and H2 indicates the second half.

The growth rate of vertical integration of hog industry chain decreased in the early stage and increased in the later stage. The growth rate of vertical integration declined in the early stage of ASF. At the beginning of ASF outbreak, the epidemic spread rapidly in China, and the hog industry was vulnerable and became the highest risk industry. If any link in the industry chain is impacted, the whole hog industry will have an impact. A large number of pigs were killed, the loss of farmers was huge, the main business of slaughter circulation companies appeared downturn, and the pig transfer policy had greatly reduced the growth rate of vertical integration of slaughter and circulation links. The process of vertical integration is stagnant. Therefore, in the early days of ASF, the pig industry as a whole appeared a downturn. Feed processing industry should prevent and control risks and accelerate the pace of vertical integration. The growth rate of vertical integration increased in the later period of ASF. With the implementation of epidemic prevention and control measures, the introduction of favorable policies for the hog industry, and the rise of pig prices, the market of the hog industry has rebounded. Under the new industrial development environment, the pig breeding industry has become a high-profit industry. As the upstream and downstream of the pig breeding industry, feed processing and slaughter processing have accelerated the layout of pig breeding.

**Fig. 2. The degree of vertical integration of each link in hog industry chain**

By comparing and analyzing the vertical integration degree of feed processing link, pig breeding link, and slaughter circulation link in hog industry chain, it is found that vertical integration degree of each link is on the rise, and that of the feed processing link is the highest (Fig. 2). Specifically, the degree of forward integration of the feed processing link is generally gradually improving, while the degree of backward integration is generally low, which indicates that the subject of hog industry chain in feed processing tends to expand the downstream industry. The backward integration degree of pig breeding is higher than forward integration, which indicates that the subject of hog industry chain in pig breeding tends to expand the upstream industry. Forward integration degree of slaughter circulation is higher than backward integration, which indicates that the subject of hog industry chain in slaughter circulation tends to expand the downstream industry. The forward integration of feed processing and slaughter circulation is higher than backward integration. Feed processing link upstream only feed raw material industry, and the planting industry has the characteristics of high risk, high investment and long period, so feed processing enterprises tend to forward integration. Feed industry in China has developed rapidly, the industry concentration has steadily improved, and the strength of enterprises has gradually increased. Existing leading enterprises have adopted the vertical integration development model. In contrast, the scale of pig breeding in China is insufficient, the concentration of slaughtering and circulation industry is low for a long time, and industry vertical integration develops slowly. Slaughter circulation downstream only has terminal retail industry. It has pricing power and high profit, so slaughtering and circulation companies tend to develop forward integration. Under the impact of ASF, the introduction of pig welfare policy and the rising of pig price increased the growth rate of vertical integration between feed processing and pig breeding (Fig. 3).

**Fig. 3. The growth rate of the vertical integration of each link**

**4. Empirical analysis**

**4.1. Variable selection**

According to the above theoretical analysis, the variables selected in this paper are mainly divided into three parts.

**4.1.1. Transaction Cost**

(1) Asset specificity

The asset specificity is positively correlated with the degree of vertical integration [37]. The assets of the hog industry are highly specialized, unable to be used for multiple purposes, and the capital conversion rate is very low. The subject of hog industry chain adopts a closer vertical integration of the industry chain to reduce operating losses when the asset specificity is high, and ultimately improve the vertical integration of the subject of hog industry chain. Current asset specificity ($Spe$) and previous asset specificity ($Spe-1$) are selected as specific indicators.

Asset specificity is expressed by the proportion of the current or previous fixed assets of the enterprise in the total assets of the enterprise

1) Current asset specificity

$Specificity=\frac{Fixedassets}{assets}$	(3)

2) Previous asset specificity

$Specificity_{-1}=\frac{Fixedassets_{-1}}{assets_{-1}}$	(4)

(2) Transaction uncertainty

In the hog industry, the pork price fluctuates greatly, which makes many breeding subjects enter and exit frequently, leading to the uncertainty of raw material supply of many slaughtering and processing subjects. Therefore, the higher the change rate of business income and business cost is, the higher the transaction uncertainty is, the higher the degree of vertical integration is. The uncertainty of operating income ($sale$) and the uncertainty of operating expenditure cost ($cost$) are used.

$Sale=\frac{income_{t}-income_{t-1}}{income_{t}}$	(5)

$Cost=\frac{{cost}_{t}-{cost}_{t-1}}{{cost}_{t}}$	(6)

$income_{t}$ is the main business income during the current period, $income_{t-1}$ is the main business income during the previous period, ${cost}_{t}$ is the main business cost during the current period, ${cost}_{t-1}$ is the main business cost during the previous period.

(3) Transaction frequency

Trading frequency refers to the number of transactions in a certain period. With each increase in the number of transactions, the relevant bargaining, signing, and other transaction costs increase a bit. In the production process of the pig in China, the transaction frequency of different links is different, resulting in different transaction costs and vertical integration of industry chains to different degrees.

1)Liquidity asset turnover ($Liquidity$). The higher turnover rate of current assets means the higher transaction frequency of the subject, which towards vertical integration to reduce costs.

$Liquidity=\frac{{income}_{t}*2}{{current}_{begin}-{current}_{end}}$	(7)

${current}_{begin}$ is the current assets at the beginning of the period, ${current}_{end}$ is the current assets at the end of the period.

2) Market share ($Market$). The higher the market demand for the main products of hog industry chain is, the more frequent the transaction with the upstream and downstream hog industry chain subjects is. In addition, because the relationship between the industry chain is not optimized, the subject chooses upstream or downstream integration to control transaction costs when the transaction cost is high (Table 3). Market share is expressed by main business income.

The market share variable is expressed by the ratio of the main business income of the enterprise to the income of all enterprises in this link.

$Market= Main business income$	(8)

Table 3. Variable descriptive statistics

| Variable | Max. | Min. | Average |
| --- | --- | --- | --- |
| Explained variable | | | |
| $V_{\mathrm{forward}}$ | 0.275 | 0 | 0.0255 |
| $V_{\mathrm{backward}}$ | 0.3041 | 0 | 0.0808 |
| Explanatory variables | | | |
| Spe | 0.5421 | 0.1052 | 0.3186 |
| Spe-1 | 0.5421 | 0.1222 | 0.3336 |
| Sale | 1.334 | -0.362 | 0.1786 |
| Cost | 1.2897 | -0.3802 | 0.1683 |
| System | 24.03 | 2.03 | 8.8794 |
| Size | 1.9516 | -0.3656 | 0.1167 |
| Age | 22 | 1 | 9.7458 |
| Liquidity | 6.0573 | 0.1624 | 2.1088 |
| Market | 0.4312 | 0.0013 | 0.0847 |

**4.1.2. Legal System Environment**

The better the legal system environment ($System$) is, the lower the degree of vertical integration is. The worse the regional legal system environment is, the greater the default risk faced by the subject of hog industry chain is, and the higher the transaction cost and bargaining cost are. Therefore, when the legal environment is poor, the subject merges the production originally organized by the market into hog industry chain to save transaction costs and reduce the risk. We used the legal environment index of the province where the sample company is registered^[1]^.

**4.1.3. Control Variable**

Enterprise ability. The ability of hog enterprises impacts vertical integration by affecting the competitive advantage of the subject. The characteristics of subjects, production and operation characteristics, and production scale also affect their behavioral choices and cause transaction uncertainty. When the subject has scarce and low alternative resources and capabilities, they choose to extend it to the relevant business areas through the vertical integration to obtain profits. Enterprise ability includes enterprise size ($Size$)and enterprise age ($\mathrm{Age}$)^[2]^. The former is expressed by the change rate of the total employees of the enterprise, and the latter is expressed by the establishment time of the enterprise as of 2019.

$Age=2019-Established+1$	(9)

$Size=\frac{{employes}_{t}-{employes}_{t-1}}{{employes}_{t-1}}$	(10)

To prevent the correlation between explanatory variables and make the results deviate, SPSS was used to test the multicollinearity of the above explanatory variables. According to table 4, VIF<0.1, tolerance > 0.1 condition index < 30, it can be seen that the explanatory variables do not have multiple collinearities, and can be further regression analyzed.

Table 4. Explanatory variable multicollinearity test

| Model | Forward | | | Backward | | |
| --- | --- | --- | --- | --- | --- | --- |
| Variable | tolerance | VIF | condition index | tolerance | VIF | condition index |
| Cons. |  |  | 1 |  |  | 1 |
| Spe | 0.247 | 4.055 | 2.246 | 0.247 | 4.055 | 2.246 |
| Spe-1 | 0.216 | 4.622 | 2.791 | 0.216 | 4.622 | 2.791 |
| Sale | 0.223 | 4.474 | 3.246 | 0.223 | 4.474 | 3.246 |
| Cost | 0.239 | 4.181 | 5.03 | 0.239 | 4.181 | 5.03 |
| Liquidity | 0.502 | 1.994 | 5.278 | 0.502 | 1.994 | 5.278 |
| System | 0.451 | 2.216 | 8.267 | 0.451 | 2.216 | 8.267 |
| Market | 0.639 | 1.565 | 13.551 | 0.639 | 1.565 | 13.551 |
| Size | 0.875 | 1.143 | 20.652 | 0.875 | 1.143 | 20.652 |
| Age | 0.555 | 1.802 | 23.783 | 0.555 | 1.802 | 23.783 |

**4.2??. Model Design**

Based on the previous theoretical analysis, the breakpoint regression and Tobit model were used to estimate the impact of ASF on the vertical integration of hog industry chain.

(1) Breakpoint regression model

At the beginning of 2018, ASF broke out. Assuming that the outbreak time is set as a breakpoint, the indicators before and after the outbreak are statistically measured by breakpoint regression analysis, and the applicability of the model is judged according to the actual situation. If the breakpoint of the model exists significantly: the vertical integration degree of hog industry chain jumps before and after the outbreak, it indicates that ASF has a greater impact on the vertical integration degree of hog industry chain. Therefore, this paper uses the breakpoint regression model to conduct an empirical study on the statistical measurement of the impact of ASF on the vertical integration of hog industry chain.

Set the breakpoint regression model as follows:

$Y_{\iota}=\alpha+\beta D_{\iota}+G(X_{\iota})+\varepsilon_{\iota}$	(11)

$Y_{i}$ is the result variable, the subscript ι represents the ιth sample, G ( $X_{\iota}$ ) represents a variety of factors that affect the result variable,$\varepsilon_{\iota}$ is a random disturbance term, and $D_{i}$is the treatment variable that indicates whether it is affected by ASF, which can be expressed as Equations (12)

$D_{\iota}=\left\{ \begin{aligned} 0, IF\iota\leq0 \\ 1, IF\iota\geq0 \end{aligned} \right.$	(12)

The formula shows that the processing variable $D_{\iota}$ is a non-continuous function affected by ASF. The breakpoint regression model is used to estimate the effect of processing variable $D_{\iota}$ on the result variable $Y_{\iota}$. In this paper, the data of the integration degree of hog industry chain before 2018 are used as the control group, and the data after 2018 are used as the experimental group. In the observation sample, 0 is taken as the dividing point ( $D_{\iota}$= 1, indicating that the result variable is affected. $D_{\iota}$ = 0 indicates that the result variable is not affected.)

(2) Basic models of forward integration and backward integration

$V_{\mathrm{forward}}=\partial_{0}+\partial_{1}Specifity+\partial_{2}\mathrm{Specifity}_{-1}+\partial_{3}sale-\partial_{4}cost+\partial_{5}Frequency+\partial_{6}System+\partial_{7}Market+\partial_{8}Size+\partial_{9}Age+\sigma$	(13)

$V_{\mathrm{backward}}=\partial_{0}+\partial_{1}Specifity+\partial_{2}\mathrm{Specifity}_{-1}+\partial_{3}sale-\partial_{4}cost+\partial_{5}Frequency+\partial_{6}System+\partial_{7}Market+\partial_{8}Size+\partial_{9}Age+\sigma$	(14)

**5. Results**

**5.1. Breakpoint Regression Results**

The breakpoint regression showed that there was a breakpoint in the backward integration of hog industry chain. The estimation of lwald200 was significantly negative at 10% level, indicating that ASF significantly inhibited the backward integration, and the backward integration showed a significant downward jump after the occurrence of ASF (Fig. 4). From Table 5, the estimation of lwald50 was significantly positive at the 1% level, indicating that ASF significantly promoted the forward integration of hog industry chain. The forward integration has an obvious upward jump after ASF (Fig. 5). ASF has accelerated the forward integration of the hog industry in China.

**Fig. 4. The breakpoint regression of backward integration graph**

The breakpoint regression shows that there is a breakpoint in the model test of the backward integration of hog industry chain affected by ASF (Table 5).

Table 5. The breakpoint regression of forward integration and backward integration result

| Result | | Coef. | Std. Err. | z | P>z |
| --- | --- | --- | --- | --- | --- |
| V _forward_ | lwald50 | 0.0001197 | 0.0440993 | 0 | 0.998 |
| V _backward_ | lwald200 | -0.0019852 | 0.0195356 | -0.1 | 0.919 |

Breakpoint regression was used to verify the backward and forward integration degree of hog industry chain. The findings show that the indicators meet the model assumptions, which are consistent with the trend of economic phenomena, and can capture the characteristics of cliff-like changes in the vertical integration of hog industry chain.

**Fig. 5. The breakpoint regression of forward integration graph**

**5.2. Empirical Results of Vertical Integration of Hog Industry Chain**

The backward integration of hog industry chain results showed that the previous asset specificity and legal system environment had a significant positive correlation, and the market demand was negatively correlated before the outbreak of ASF. The higher the previous asset specificity was, the higher the degree of backward integration of hog industry chain was.

Compared with before the ASF, the determinants of backward integration have no noticeable change (Table 6). Specifically, The higher the legal system environment was, the higher the backward integration degree was. A higher legal system environment means a better market trading environment, the subject will rapidly develop and increase the frequency of transactions Although the higher legal system environment avoided the cost of default for the subject of hog industry chain, the higher the transaction frequency was, the higher the bargaining cost was. Therefore, when the legal system environment is higher, the degree of backward integration of enterprises is higher. The higher the market demand was, the lower the degree of backward integration was. Along with the enhancement of market demand, the scale of product processing link of the industry chain subject gradually enlarged. When the processing link reaches the scale economy effect, this link would be separated from the original industry chain subject to improve the specialization degree of the industry chain. Therefore, the backward integration of the industry chain decreased with the increase of market demand.

Table 6. The analysis results of the backward integration

| V _backward_ | Model 1 | Model 2 |
| --- | --- | --- |
| Spe | 0.18  1.610 | 0.17  1.350 |
| Spe-1 | 0.24  2.210^(**)^ | 0.27  1.7 |
| Sale | 0.01  0.210 | 0.03  0.41 |
| Cost | 0.004  0.090 | 0.05  0.93 |
| Liquidity | 0.01  1.220 | 0.01  0.73 |
| System | 0.01  7.660^(***)^ | 0.01  4.06^(***)^ |
| Market | -0.08  -3.420^(***)^ | -0.16  -3.24^(***)^ |
| Size | -0.01  -0.490 | 0.01  0.17 |
| Age | -0.003  -1.340 | 0.004  1.14 |
| Constant | -0.14  -4.110^(***)^ | -0.26  -3.58^(***)^ |

Note: (1) Model 1 is the regression result for 2012-2017 and model 2 is the regression result for 2018-2019.

(2) *, * *, * * * respectively represent 10%, 5%, 1% significance.

In the forward integration of hog industry chain, the establishment time of the subject had a positive correlation, and the asset specificity of the previous period was significantly negatively correlated before the outbreak of ASF. After the outbreak of ASF, the regression results showed that income uncertainty, legal system environment and market demand had a significant negative correlation with the forward integration of hog industry chain. Expenditure uncertainty, transaction frequency, and the establishment time of the subject were significantly positively correlated with the forward integration of the subject (Table 7).

Table 7. The analysis results of the forward integration

| V _forwaed_ | Model 1 | Model 2 |
| --- | --- | --- |
| Spe | -0.28  -1.05 | 0.1  0.7 |
| Spe-1 | -0.59  -2.43^(**)^ | 0.21  1.19 |
| Sale | -0.05  -0.87 | -0.67  -5.32^(***)^ |
| Cost | -0.10  -1.74 | 0.37  3.45^(**)^ |
| Liquidity | -0.002  -0.23 | 0.13  ^(***)^ |
| System | 0.005  0.41 | -0.02  -4.37^(***)^ |
| Market | -1.03 | -0.65  -5.69^(***)^ |
| Size | 0.02  1.05 | -0.07  -0.96 |
| Age | 0.01  2.53^(**)^ | 0.03  3.72^(***)^ |
| Constant | 0.80  3.67^(***)^ | -0.51  -3.07^（**)^ |

Note: (1) Model 1 is the regression result for 2012-2017 and model 2 is the regression result for 2018-2019.

(2) *, * *, * * * respectively represent 10%, 5%, 1% significance.

The longer the establishment time was, the higher the forward integration degree of hog industry chain was. The higher the long-term asset specificity was, the lower the forward integration of hog industry chain was. In hog industry chain, feed processing and slaughter circulation were mainly integrated. The asset specificity of these two links was very high, so the industry barrier area and internal monopoly were high. Therefore, the higher the asset specificity is, the higher the monopoly profit is. Compared with entry of low-profit industries, the subject of the industry chain is more willing to maintain production and operation in the current link.

Income uncertainty, legal system environment, and market demand have a significant negative correlation with the forward integration. Expenditure uncertainty, transaction frequency, and the establishment time of the subject are significantly positively correlated with the forward integration, legal environment, expenditure uncertainty, transaction frequency, and establishment time. The higher the expenditure uncertainty and transaction frequency are, the higher the degree of forward integration is. The higher legal environment is, the lower the forward integration is. Market demand is consistent with the impact on the forward integration before the impact of ASF. The lower the change rate of business income is, the higher the forward integration is. The low change rate of operating income means that the main production and operation of hog industry chain is stable, and the production scale reaches economies of scale. Under the impetus of economies of scale, the more stable the operating income is, the more hog industry chain is inclined to carry out a forward integration.

**6. Discussion and Conclusion**

**6.1. Discussion**

This paper studies the vertical integration of hog industry chain from the perspective of stable supply. The study found that the degree of vertical integration of hog industry chain has been increasing year by year in China. The outbreak of ASF has disrupted the operation of the feed, breeding and slaughtering, and processing links in hog industry chain. The feed processing link pays more attention to forward integration. Under the guidance of the actual market demand, feed companies choose to use feed with high digestion and high disease resistance to ensure the safety of pig breeding and reduce production costs. The degree of backward integration in the breeding link is higher than forward integration. Listed companies have large-scale farming, and their marginal cost elasticity is small. They will choose more to cooperate with upstream raw material suppliers, and widen the gap with the industry average cost. In the context of the policy of prohibiting the inter-provincial transfer of hogs, slaughtering companies are more likely to choose forward integration to avoid overstocking and ensure local pork supply.

ASF has promoted the forward integration of the hog industry chain in China, which results from the fact that forward integration enables companies to better control the retail price and to respond more effectively to the changes in market demand changes [38]. According to the transaction cost theory, transaction cost, transaction frequency, and transaction uncertainty will affect the way companies choose vertical integration. Therefore, this paper further explores the determinants that affect the adoption of forward and backward integration by listed hog companies. The empirical results show that income uncertainty, legal system environment, and market demand have a significant negative correlation with the forward integration of the pig industry chain under the impact of ASF. It is also confirmed that due to the impact of the epidemic, the price of live pigs fluctuates greatly, consumer doubts arise, and market demand is weak. In this context, hog industry chain is suitable for forward integration. Policy support for the forward integration of hog industry chain in areas with relatively low legal and institutional environments should be increased.

This study also has several limitations. In order to make the research focus more specific, we do not analyze the integration methods in other directions. In addition, due to the short time since the entry of ASF into China, it is difficult to obtain relevant information, and the research time is limited. This paper fails to summarize the experience of other countries regarding the vertical integration of hog industry chain, and the follow-up will be an important research direction.

**6.2. Conclusions**

After the outbreak of ASF, hog industry and pork supply in China is facing new development challenges. Under the normalization of ASF, vertical integration can reduce transaction costs of pig companies, and stabilize supply in hog industry chain, which has become an important way to ensure a stable supply of pigs. The main conclusion is as follows:

(1) The degree of vertical integration of the hog industry chain in China has been increasing year by year. Influenced by the ASF epidemic, the degree of vertical integration of the subject in different links fluctuates in different situations. The backward integration of the pig breeding link is higher than the forward integration, and the forward integration of the feed processing link and slaughter circulation link is higher than the backward integration. The integration of feed and breeding links to a certain extent reflects the integration of the pig industry within the primary industry. The vertical integration method based on the combination of planting and breeding not only ensures food safety but also promotes the sustainable development of the hog industry. ASF has promoted changes in domestic live pig transportation policies, and the transformation of live pig transportation into chilled meat transportation has stimulated forward integration in the slaughter circulation link.

(2) Under the impact of ASF, the vertical integration degree of hog industry chain jump, while ASF inhibit the backward integration degree of hog industry chain and promote the forward integration. Hog industry chain is taking the forward integration, increasing the supply of pork by improving the control over the supply of upstream raw materials.

(3) Under the impact of ASF, the determinants of backward integration have not changed significantly. The higher the legal system environment is, the better the market trading environment is. The subject increases the transaction frequency, and backward integration will increase. The increase in market demand will increase the scale of product processing links for the subject. When the processing link reaches economies of scale, this link will be separated from the subject of the original industry chain, and increase the specialization of the companies. Therefore, the degree of backward integration will decrease with the increase in transaction frequency.

(4) Under the impact of ASF, the determinants of the forward integration have increased, such as income uncertainty, legal system environment, market demand, expenditure uncertainty, transaction frequency and establishment time. These factors have a significant impact on the vertical integration of the pig industry chain. The system environment inhibits the forward integration, the lower the rate of change in operating income means that the production and operation of hog industry chain are stable, and the scale of production has reached economies of scale. Driven by the economies of scale, the more stable the operating income is, the more hog industry chain is inclined to carry out the forward integration and guarantee pork quality and stable supply.

**Supporting information**

Table S1. [Influence](D:/software/Youdao/Dict/8.10.3.0/resultui/html/index.html#/javascript:;) [factor](D:/software/Youdao/Dict/8.10.3.0/resultui/html/index.html#/javascript:;)s.xlsx

**Acknowledgments**

We would like to thank the editors and two reviewers whose comments helped to greatly improve this manuscript.

**Author Contributions**

Conceptualization, Gangyi Wang; Methodology, Jingjing Wang; Software, Jingjing Wang; Validation, Change Zhao; Formal Analysis, Siyu Chen and Jingjing Wang; Resources, Siyu Chen; Data Curation, Siyu Chen and Change Zhao; Writing—Original Draft Preparation, Siyu Chen and Jingjing Wang; Writing—Review and Editing, Gangyi Wang and Jingjing Wang; Funding Acquisition, Gangyi Wang.

**Funding**

This work was funded by the Natural Science Foundation of Heilongjiang Province in China (Research on Capital Stress and Guidance of Pig Industry in Heilongjiang Province under Environmental Regulation, Project number LH2019G002) and the Chongqing Talents Program in China.

**Institutional Review Board Statement**

Not Applicable.

**Informed Consent Statement**

Informed consent was obtained from all subjects involved in the study.

**Data Availability Statement**

The data presented in this study are available from the corresponding author.

**Conflicts of Interest**

The authors declare no conflicts of interest.

**References**

**1.**	Herold P, Roessler R, Willam A, Momm H, Valle Zárate A. Breeding and supply chain systems incorporating local pig breeds for small-scale pig producers in Northwest Vietnam. Livestock Science. 2010; 129(1-3): 63-72. <https://doi.org/10.1016/j.livsci.2010.01.004>.

**2.**	Jackson P, Guy J, Edwards SA, Sturm B, Bull S. Application of dynamic thermal engineering principles to improve the efficiency of resource use in UK pork production chains. Energy & Buildings. 2017; 139(MAR.): 53-62.

**3.**	Estampe D, Lamouri S, Paris JL, Brahim-Djelloul S. A framework for analysing supply chain performance evaluation models. International Journal of Production Economics. 2013; 142(2): 247-258.

**4.**	Lambert DM, Enz MG. Issues in Supply Chain Management: Progress and potential. Industrial Marketing Management. 2017; 62: 1-16. <https://doi.org/https://doi.org/10.1016/j.indmarman.2016.12.002>.

**5.**	Wu P, Jin X, Ma L. China’s regional Economic Development Gap from the Perspective of Numerical Distribution and Spatial Distribution (1952–2008). Economic Science. 2010; 5: 46-58.

**6.**	Mena C, Humphries A, Choi TY. Toward a theory of multi‐tier supply chain management. Journal of Supply Chain Management. 2013; 49(2): 58-77.

**7.**	Choi TY, Hong Y. Unveiling the structure of supply networks: case studies in Honda, Acura, and DaimlerChrysler. Journal of Operations Management. 2002; 20(5): 469-493.

**8.**	Wang Y, Wang J, Wang X. COVID-19, supply chain disruption and China’s hog market: a dynamic analysis. China Agricultural Economic Review. 2020.

**9.**	Pu M, Zhong Y. Rising concerns over agricultural production as COVID-19 spreads: Lessons from China. Global Food Security. 2020; 26: 100409.

**10.**	Mason-D’Croz D, Bogard JR, Herrero M, Robinson S, Sulser TB, Wiebe K, et al. Modelling the global economic consequences of a major African swine fever outbreak in China. Nature Food. 2020; 1(4): 221-228.

**11.**	Niemi JK. Impacts of African swine fever on pigmeat markets in Europe. Frontiers in Veterinary Science. 2020; 7: 634.

**12.**	Taylor RA, Condoleo R, Simons RR, Gale P, Kelly LA, Snary EL. The risk of infection by African swine fever virus in European swine through boar movement and legal trade of pigs and pig meat. Frontiers in veterinary Science. 2020; 6: 486.

**13.**	Beltran-Alcrudo D, Gallardo M, Kramer S, Penrith M, Kamata A, Wiersma L. African swine fever: detection and diagnosis: Food and Agriculture Organization of the United Nations (FAO); 2017.

**14.**	Woonwong Y, Do Tien D, Thanawongnuwech R. The future of the pig industry after the Introduction of African swine fever into Asia. Animal Frontiers. 2020; 10(4): 30-37. <https://doi.org/10.1093/af/vfaa037>.

**15.**	Ma M, Wang HH, Hua Y, Qin F, Yang J. African swine fever in China: Impacts, responses, and policy implications. Food Policy. 2021: 102065.

**16.**	Woonwong Y, Tien DD, Thanawongnuwech R. The Future of the Pig Industry After the Introduction of African swine fever into Asia. Animal Frontiers. 2020; 10(4): 30-37.

**17.**	Nadal-Roig E, Plà-Aragonès LM, Alonso-Ayuso A. Production planning of supply chains in the pig industry. Computers and Electronics in Agriculture. 2019; 161: 72-78.

**18.**	Sant'Anna AC, Bergtold JS, Shanoyan A, Granco G, Caldas MM. Examining the relationship between vertical coordination strategies and technical efficiency: Evidence from the Brazilian ethanol industry. Agribusiness. 2018; 34(4): 793-812.

**19.**	Peyrefitte J, Golden PA. Vertical integration and performance in the United States computer hardware industry. International Journal of Management. 2004; 21(2): 246.

**20.**	Martins FM, Trienekens J, Omta O. Implications of horizontal and vertical relationships on farmers performance in the Brazilian pork industry. Livestock Science. 2019; 228: 161-169. <https://doi.org/10.1016/j.livsci.2019.08.013>.

**21.**	Abdul‐Rahaman A, Abdulai A. Vertical coordination mechanisms and farm performance amongst smallholder rice farmers in northern Ghana. Agribusiness. 2020; 36(2): 259-280.

**22.**	Dong DD, Moritaka M, Liu R, Fukuda S. Restructuring toward a modernized agro-food value chain through vertical integration and contract farming: the swine-to-pork industry in Vietnam. Journal of Agribusiness in Developing and Emerging Economies. 2020; ???.

**23.**	Ouma E, Ochieng J, Dione M, Pezo D. Governance structures in smallholder pig value chains in Uganda: constraints and opportunities for upgrading. Journal? 2017; ???.

**24.**	East I, Davis J, Sergeant E, Garner M. Structure, dynamics and movement patterns of the Australian pig industry. Australian Veterinary Journal. 2014; 92(3): 52-57.

**25.**	Klein B, Crawford RG, Alchian AA. Vertical integration, appropriable rents, and the competitive contracting process. The Journal of Law and Economics. 1978; 21(2): 297-326.

**26.**	Cheung SN. The contractual nature of the firm. The Journal of Law and Economics. 1983; 26(1): 1-21.

**27.**	Oliver W. The economic institutions of capitalism. Journal of Economic. 1985; ???.

**28.**	Williamson OE. Transaction-cost economics: the governance of contractual relations. The journal of Law and Economics. 1979; 22(2): 233-261.

**29.**	Huang D, Liu L. Micro-determinants of vertical integration: Evidence from China. Asia Pacific Journal of Management. 2014; 31(2): 377-396.

**30.**	Rey P, Tirole J. A primer on foreclosure. Handbook of industrial organization. 2007; 3: 2145-2220.

**31.**	Demsetz H. The theory of the firm revisisted. JL Econ & Org. 1988; 4: 141.

**32.**	Gulen H, Ion M. Policy uncertainty and corporate investment. The Review of Financial Studies. 2016; 29(3): 523-564.

**33.**	Grossman SJ, Hart OD. The costs and benefits of ownership: A theory of vertical and lateral integration. Journal of Political economy. 1986; 94(4): 691-719.

**34.**	Loasby BJ. Organisational capabilities and interfirm relations. Metroeconomica. 1994; 45(3): 248-265.

**35.**	Fan JP, Huang J, Morck R, Yeung B. Vertical integration, institutional determinants and impact: Evidence from China. National Bureau of Economic Research, 2009.

**36.**	Joskow PL. Vertical Integratio. The Antitrust Bulletin. 2010; 55(3): 545-586.

**37.**	Argyres NS, Zenger TR. Capabilities, transaction costs, and firm boundaries. Organization Science. 2012; 23(6): 1643-1657.

**38.**	Lin Y-T, Parlaktürk AK, Swaminathan JM. Vertical Integration under Competition: Forward, Backward, or No Integration? Production and Operations Management. 2014; 23(1): 19-35. <https://doi.org/10.1111/poms.12030>.

1. The specific index source from 2012 to 2018 is the Marketization Index Report of China by Province ( 2018 ). The calculation of the legal system environment index in 2019 used the three-year moving average method.
2. Enterprise size is expressed by the rate of change in total employees of the enterprise, and enterprise age is expressed by the establishment time of the enterprise as of 2019.
