# Supplementary material for: Vertical integration selection of Chinese pig industry chain under African swine fever - From the perspective of stable pig supply
Source: PLoS One. 2023 Feb 23;18(2):e0280626. doi: 10.1371/journal.pone.0280626 (PMC9949627; doi:10.1371/journal.pone.0280626)
Supplement: Attachment — Submitted filename: Response to Reviewers.docx [file pone.0280626.s003.docx]

Response to Reviewers

Dear editors and reviewers,

Thank you for your valuable feedback on our manuscript entitled " **Vertical Integration Selection of Chinese Pig Industry Chain under African Swine Fever - From the Perspective of Stable Pig Supply** ". Those comments are all valuable and very helpful for revising and improving our paper, as well as the important guiding significance to our research.

We have carefully studied the comments of the editor and reviewer comments, and based on the comments, the paper has been modified as follows (the red font below is the comments of the editor and reviewers, and the black font below is the modification description):

**Responses to the comments of the editors**

**Comment-1:** Please ensure that your manuscript meets PLOS ONE's style requirements, including those for file naming.

**Response:** Thank you sincerely for this comment. We can ensure that our manuscript meets PLOS ONE's style requirements.

**Comment-2:** We note that the grant information you provided in the ‘Funding Information’ and ‘Financial Disclosure’ sections do not match. When you resubmit, please ensure that you provide the correct grant numbers for the awards you received for your study in the ‘Funding Information’ section.

**Response:** Thank you sincerely for this comment. Based on the comment of the editor, our cover letter includes the updated financial statement. Details are as follows: 'This research is supported by the Natural Science Foundation of Heilongjiang Province China (Project number LH2019G002); and the Humanities and Social Science Foundation of Ministry of education of China (Project number 21YJA790053). The funders had no role in study design, data collection and analysis, decision to publish, or preparation of the manuscript. '

**Comment-3:**  In your Data Availability statement, you have not specified where the minimal data set underlying the results described in your manuscript can be found. PLOS defines a study's minimal data set as the underlying data used to reach the conclusions drawn in the manuscript and any additional data required to replicate the reported study findings in their entirety. All PLOS journals require that the minimal data set be made fully available. For more information about our data policy, please see <http://journals.plos.org/plosone/s/data-availability.>

**Response:** Thank you sincerely for this comment. We have added “S1 Table. [Influence](D:/software/Youdao/Dict/8.10.3.0/resultui/html/index.html#/javascript:;) [factor](D:/software/Youdao/Dict/8.10.3.0/resultui/html/index.html#/javascript:;)s.xlsx” as Supporting information

**Comment-4:** Please ensure that you refer to Figures 1, in your text as, if accepted, production will need this reference to link the reader to the figure.

**Response:** Thank you sincerely for this comment. We have referred to Figures 1 in our text.

**Comment-5:** We note you have included a table to which you do not refer in the text of your manuscript. Please ensure that you refer to Table 1 in your text; if accepted, production will need this reference to link the reader to the Table.

**Response:** Thank you sincerely for this comment. We have referred to Table 1 in our text.

**Responses to the comments of Reviewer #1**

**Comment:** The present study analyzed the driving forces behind the vertical integration of the listed pig companies in China in response to African swine fever outbreaks. The work is helpful for the understanding of the recent dramatic changes of the Chinese pig Industry. However, several concerns need to be addressed. The data from the latest years of the 12 listed Chinese pig companies should be included in the analysis by more independent methods. The manuscript was poorly written and should be revised by native English speakers and agro-economists.
 **Response:** Thank you sincerely for this comment. Based on the comments of the reviewers, first of all, we added the analysis of the sample companies(Line 214-248), secondly, we added the discussion on the determinants of vertical integration of enterprises in different links in the empirical analysis section(Line456-466 ), finally, we have optimized the grammar and language.

With best regards

Yours sincerely,

Gangyi Wang

Jingjing Wang

Siyu Chen

Chang’e Zhao
